# Editorial: Novel approaches in diagnosis and management of ischemic edema in large vessel occlusion stroke, volume II

**DOI:** 10.3389/fneur.2025.1568007

**Published:** 2025-02-19

**Authors:** Gabriel Broocks, Jens Minnerup, Shervin Kamalian, Andre Kemmling

**Affiliations:** ^1^Department of Neuroradiology, HELIOS Medical Center, Campus of MSH Medical School Hamburg, Schwerin, Germany; ^2^Department of Neurology, University Hospital Lübeck, Lübeck, Germany; ^3^Department of Neuroradiology, Beth Israel Deaconess Medical Center, and Harvard Medical School, Boston, MA, United States; ^4^Department of Neuroradiology, University Marburg, Marburg, Germany

**Keywords:** stroke, thrombectomy, imaging—computed tomography, neuroprotection, ischemia—reperfusion

## Introduction

Recently, randomized controlled trials (RCTs) have confirmed that endovascular thrombectomy (EVT) significantly improves functional outcome and survival in patients with anterior stroke presenting with large early infarct lesions on standard imaging, compared to best medical treatment (BMT) alone (1–6). Additionally, it has been observed that the effect of recanalization on functional outcome is mediated by edema reduction as an important mechanism explaining the treatment effect particularly in large strokes. Still, there is only very limited proof of effective adjuvant treatment options to further reduce the impact of edema formation. The aim of this Research Topic was (1) to investigate further potential adjuvant treatment strategies for ischemic stroke and (2) to discuss novel prediction models for the assessment of secondary injury volumes directly affecting functional outcome.

## Novel prediction models and imaging techniques

The early identification of patients at risk of developing potentially malignant brain edema is of significant clinical relevance. Liu et al. systematically evaluated prediction models for post-thrombectomy brain edema in acute ischemic stroke patients. The authors observed a high risk of bias after analyzing 10 models and emphasized the need for large-scale, multicenter studies to develop robust models for real-world clinical applications (Liu et al.). Similarly, Ma et al. investigated intracranial hemorrhage as further secondary injury after stroke using a nomogram prediction model including 197 stroke patients receiving intravenous thrombolysis with alteplase. The highest diagnostic accuracy to predict secondary hemorrhage was observed for a nomogram including NIHSS, NT-pro BNP, neutrophil to lymphocyte ratio, and systolic blood pressure (Ma et al.).

A further nomogram analysis for outcome prediction in stroke patients was used by Xie et al. specifically to predict the risk for malignant cerebral edema. After inclusion of 312 patients, the developed nomogram based on LASSO-logistic regression was an accurate predictor of malignant edema showing an AUC of 0.89 (Xie et al.). Pham and Ng comprehensively discussed the currently available advanced imaging techniques for cerebral edema in ischemic stroke. Several techniques exist, however, many challenges remain that must be met before clinical application is possible.

## Novel strategies for the management of vasogenic edema after stroke

Several drugs have been tested as options for adjuvant treatment and neuroprotection in ischemic stroke over decades. Despite many promising animal studies, there is yet no effective and established treatment available to further improve functional outcome (7). Yet, a majority of these trials were including patients not undergoing successful endovascular treatment (8). The combination of reperfusion with adjuvant treatment might bring forth new strategies to improve functional outcome (8). Pan et al. presented a phase 1 clinical trial using Albumin as adjuvant treatment option, which previously showed neuroprotective effects in an animal population. The authors aimed to investigate the safety and efficacy of arterial infusion of an albumin solution after recanalization. The authors expected to provide data for subsequent studies on the arterial infusion of albumin solutions (Pan et al.). A further strategy for neuroprotection might consist of epidermal growth factor and growth hormone-releasing hexapeptide. Hernández-Bernal et al. on behalf of the (COmbined therapeUtic appRoAch durinG acute strokE) COURAGE group presented a phase I/II randomized clinical trial and concluded that the administration of these agents was safe and a phase III study may bring forth promising clinical benefits. Finally, Yan et al. discussed protective effects of butyrate on ischemic injury in animal models aiming to provide a scientific basis for the clinical application of butyrate in stroke patients. It was concluded that butyrate may exhibit a protective effect in animals relating to reduced inflammation and inhibition of apoptosis based on the analysis of nine studies (Yan et al.).

## Discussion and future challenges

Recently, the publication of the large core trials further expanded the indication for EVT to patients that were previously often excluded from treatment. Demonstrating a significant clinical benefit of vessel recanalization in patients despite already showing signs of significant lesion progression challenges adhered dogmas of treatment decision-making with implications for the treatment of ischemic stroke in general (9). As last frontiers, the effect of EVT for patients presenting with distal vessel occlusions and low NIHSS is currently being investigated in several randomized trials (10).

Nevertheless, the treatment effect of reperfusion especially in large stroke is not well-understood and requires further investigation. The degree of early edema formation in ischemic stroke, which can be determined for instance using CT densitometry, has not yet been considered as image criterion for treatment selection, which could potentially be an important modifier of the therapy effect of recanalization (11). Furthermore, secondary treatment effects like edema reduction may be the link between better functional outcome despite a large infarct. In this context, adjuvant treatment options targeting edema formation may be of interest (12). In the CHARM trial (NCT02864953), the safety and efficacy of intravenous glibenclamide to treat cerebral edema was investigated after previous studies observed that glibenclamide administration was associated with reduced edema formation and better functional outcome (13, 14). However, in patients undergoing EVT, the administration of this drug in this study was only applied after the EVT procedure and after performing a further DWI-MRI examination, which is a significant limitation, also considering that this trial was originally not designed to evaluate patients undergoing EVT. In the future, the timely administration of glibenclamide in combination with other neuroprotectants and in the context of endovascular recanalization may lead to better outcomes in ischemic stroke.
